# Crystal structure and Hirshfeld surface analysis of 5-amino-5′-bromo-2′-oxo-2,3-di­hydro-1*H*-spiro­[imidazo[1,2-*a*]pyridine-7,3′-indoline]-6,8-dicarbo­nitrile dimethyl sulfoxide disolvate

**DOI:** 10.1107/S2056989022004741

**Published:** 2022-05-10

**Authors:** Farid N. Naghiyev, Victor N. Khrustalev, Anton P. Novikov, Mehmet Akkurt, Rovnag M. Rzayev, Anzurat A. Akobirshoeva, Ibrahim G. Mamedov

**Affiliations:** a Department of Chemistry, Baku State University, Z. Khalilov str. 23, Az 1148, Baku, Azerbaijan; b Peoples’ Friendship University of Russia (RUDN University), Miklukho-Maklay St. 6, Moscow 117198, Russian Federation; c N. D. Zelinsky Institute of Organic Chemistry RAS, Leninsky Prosp. 47, Moscow 119991, Russian Federation; d Department of Physics, Faculty of Sciences, Erciyes University, 38039 Kayseri, Turkey; e ‘Composite Materials’ Scientific Research Center, Azerbaijan State Economic University (UNEC), H. Aliyev str. 135, Az 1063, Baku, Azerbaijan; f Academy of Sciences of the Republic of Tajikistan, Kh Yu Yusufbekov Pamir Biological Institute, 1 Kholdorova St, Khorog 736002, Gbao, Tajikistan; University of Durham, United Kingdom

**Keywords:** crystal structure, spiro­[imidazo[1,2-*a*]pyridine, hydrogen bonds, dimethyl sulfoxide, disorder, Hirshfeld surface analysis

## Abstract

In the title compound, C_16_H_11_BrN_6_O·2C_2_H_6_OS, the 1,2,3,7-tetra­hydro­imid­azo[1,2-*a*]pyridine ring system and the oxindole moiety are both nearly planar [maximum deviations = 0.042 (2) and 0.115 (2) Å, respectively] and their planes form a dihedral angle of 86.04 (5)° with each other. Inter­molecular N—H⋯O, C—H⋯O and C—H⋯N hydrogen bonds link mol­ecules in the crystal through the O atoms of the solvent mol­ecules, generating a three-dimensional network. A Hirshfeld surface analysis was performed to further analyse the inter­molecular inter­actions.

## Abstract 2

Inter­molecular N—H⋯O, C—H⋯O and C—H⋯N hydrogen bonds form a three-dimensional network in the crystal, connecting mol­ecules through the O atoms of solvent mol­ecules.

## 1. Chemical context

C—C and C—N bond-forming reactions represent a significant synthetic class because they play critical roles in various applications in different fields of chemistry (Yadigarov *et al.*, 2009 ▸; Abdelhamid *et al.*, 2011 ▸; Yin *et al.*, 2020 ▸; Khalilov *et al.*, 2021 ▸). Nitro­gen heterocycles, particularly those including the spiro­[imidazo[1,2-*a*]pyridine] moiety, play a key role in medi­cinal chemistry (Han *et al.*, 2008 ▸; Mamedov *et al.*, 2020 ▸; Samaneh *et al.*, 2021 ▸). The conjugate addition to oxo­in­dol­inylidenemalono­nitriles has been well studied in simple two-component reactions with respect to producing spiro derivatives (Lu *et al.*, 2012 ▸; Jun *et al.*, 2019 ▸). We have previously reported the three-component reaction of 2-(2-oxoindolin-3-yl­idene)malono­nitrile with malono­nitrile and ethyl­enedi­amine which resulted in 5-amino-2′-oxo-2,3-di­hydro-1*H*-spiro­[imidazo[1,2-*a*]pyridine-7,3′-indoline]-6,8-dicarbo­nitrile (Magerramov *et al.*, 2018 ▸). In the framework of our ongoing structural studies (Naghiyev *et al.*, 2020 ▸, 2021*a* ▸,*b* ▸), herein the crystal structure and Hirshfeld surface analysis of 5-amino-5′-bromo-2′-oxo-2,3-di­hydro-1*H*-spiro­[imidazo[1,2-*a*]pyridine-7,3′-indoline]-6,8-dicarbo­nitrile, (1), is reported.

## 2. Structural commentary

**Figure 1:**
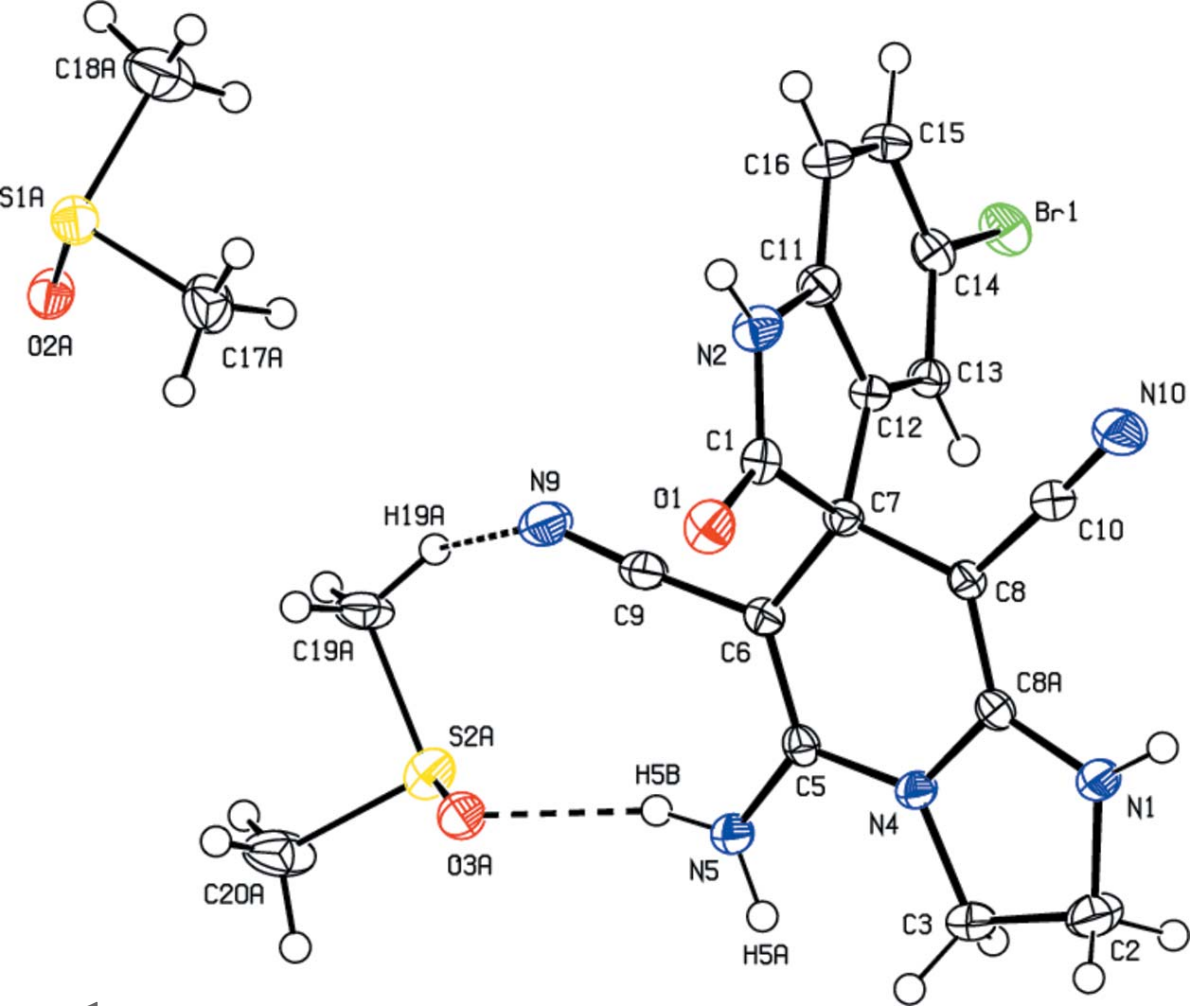
The title mol­ecule with the labelling scheme and displacement ellipsoids drawn at the 50% probability level. The minor components of the disorder are not shown.

In the title compound, (1) (see Scheme[Chem scheme1] and Fig. 1 ▸), the 1,2,3,7-tetra­hydro­imidazo[1,2-*a*]pyridine ring system (N1/N4/C2/C3/C5–C8/C8*A*) and the oxindole moiety (O1/N2/C1/C7/C11–C16) are nearly planar, with maximum deviations of 0.042 (2) Å for C3 and 0.115 (2) Å for O1. These ring systems make a dihedral angle of 86.04 (5)° with each other. The cyano (–C≡N) and amine (NH_2_) groups form an inter­molecular hydrogen bond with one dimethyl sulfoxide (DMSO) group, giving an *S*(10) motif (Bernstein *et al.*, 1995 ▸) (Table 1 ▸). 
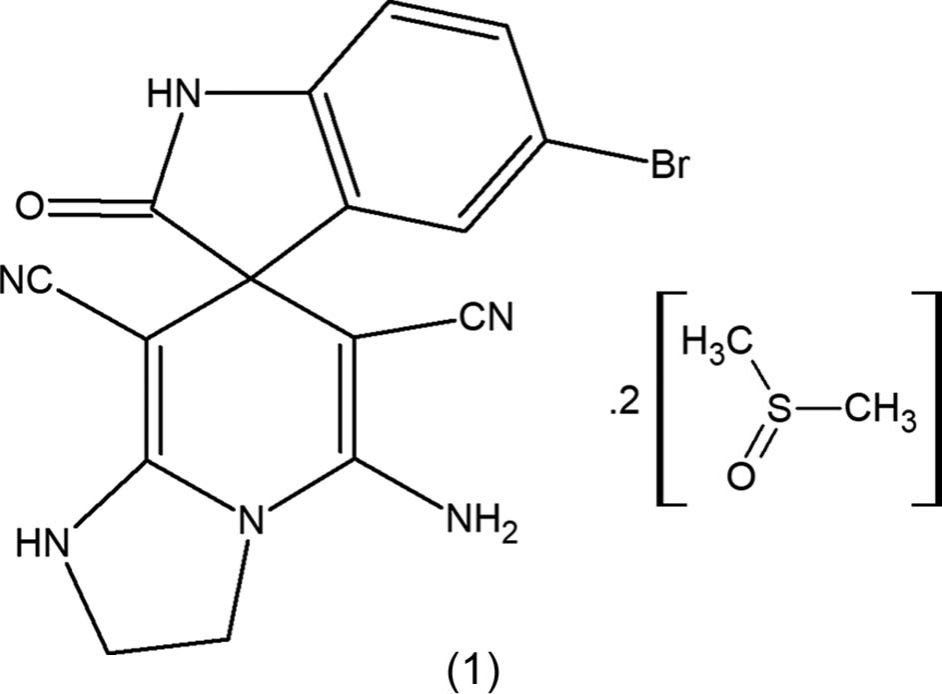


## 3. Supra­molecular features and Hirshfeld surface analysis

**Figure 2:**
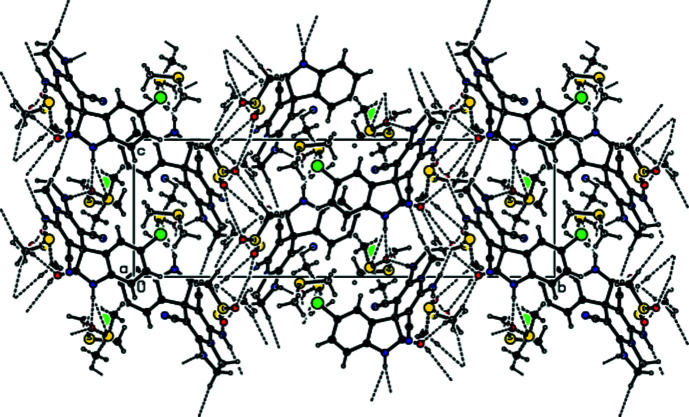
A view of the mol­ecular packing of (1) along the *a*-axis direction.

In the crystal, mol­ecules are linked through the O atoms of the DMSO solvent mol­ecules by inter­molecular N—H⋯O and C—H⋯O hydrogen bonds which, together with C—H⋯N hydrogen bonds, form a three-dimensional (3D) network (Table 1 ▸ and Fig. 2 ▸). The π-cloud of the C8*A*—N1 bond (which has some multiple-bond character) acts as an electron donor to Br1 in a kind of ‘halogen bond’, with a Br1⋯C8*A*(−*x* + 1, −*y* + 1, −*z*) distance of 3.284 (2) Å.

**Figure 3:**
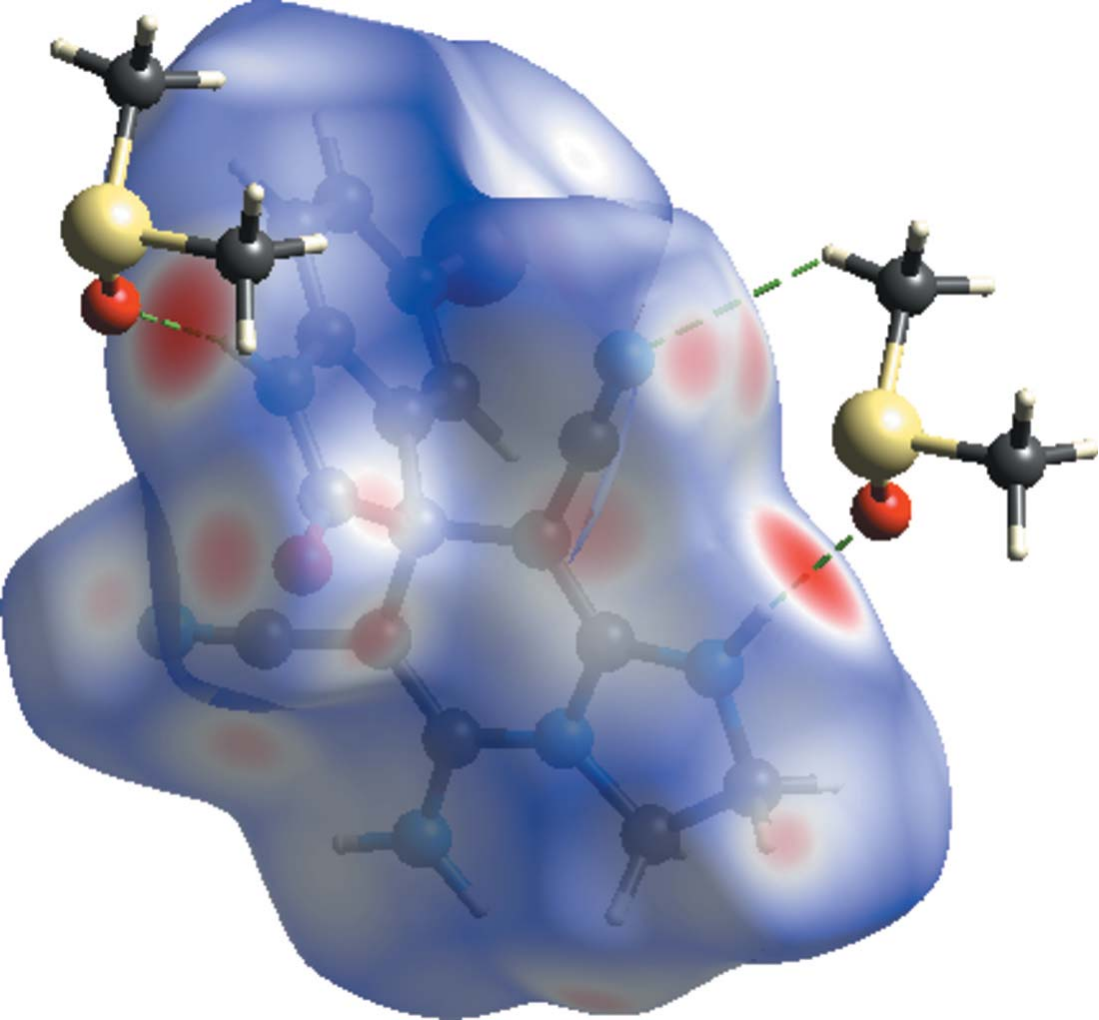
View of the 3D Hirshfeld surface of (1) plotted over *d*_norm_ in the range from −0.6206 to 1.3180 a.u.

The Hirshfeld surfaces were calculated and the two-dimensional (2D) fingerprint plots generated using *CrystalExplorer* (Version 17.5; Turner *et al.*, 2017 ▸). Fig. 3 ▸ shows the 3D Hirshfeld surface of (1) with *d*_norm_ (normalized contact distance) plotted over the range from −0.6206 to 1.3180 a.u. The inter­actions given in Table 1 ▸ play a key role in the mol­ecular packing of (1).

**Figure 4:**
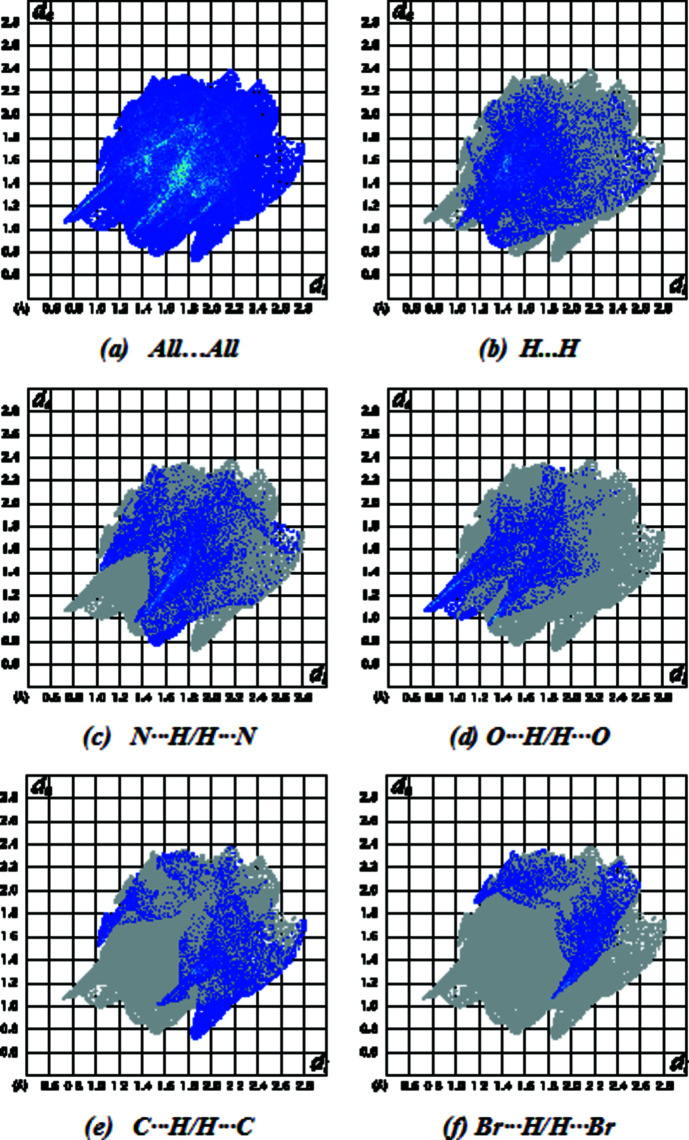
The full 2D fingerprint plots for (1), showing (*a*) all inter­actions, and delineated into (*b*) H⋯H, (*c*) N⋯H/H⋯N, (*d*) O⋯H/H⋯O, (*e*) C⋯H/H⋯C and (*f*) Br⋯H/H⋯Br inter­actions. The *d*_i_ and *d*_e_ values are the closest inter­nal and external distances (in Å) from given points on the Hirshfeld surface contacts.

The overall 2D fingerprint plot for (1) is given in Fig. 4 ▸(*a*), and those delineated into H⋯H, N⋯H/H⋯N, O⋯H/H⋯O, C⋯H/H⋯C and Br⋯H/H⋯Br contacts are shown in Figs. 4 ▸(*b*)–(*f*). The percentage contributions to the Hirshfeld surfaces from the various inter­atomic contacts are as follows: H⋯H [Fig. 4 ▸(*b*); 27.1%], N⋯H/H⋯N [Fig. 4 ▸(*c*); 23.8%], O⋯H/H⋯O [Fig. 4 ▸(*d*); 15.7%], C⋯H/H⋯C [Fig. 4 ▸(*e*); 13.2%] and Br⋯H/H⋯Br [Fig. 4 ▸(*f*); 10.2%]. Other minor contributions to the Hirshfeld surface are from Br⋯C/C⋯Br (3.9%), Br⋯N/N⋯Br (2.0%), C⋯C (1.5%), S⋯C/C⋯S (0.8%), S⋯H/H⋯S (0.6%), S⋯N/N⋯S (0.4%), O⋯N/N⋯O (0.4%) and Br⋯O/O⋯Br (0.3%).

## 4. Database survey

A search of the Cambridge Structural Database (CSD, Version 5.42, update of September 2021; Groom *et al.*, 2016 ▸) for the 5-bromo-1,3-di­hydro-2*H*-indol-2-one unit of (1) gave 87 hits. The three compounds most resembling (1) are (**I**) (COGQAS; Nagalakshmi *et al.*, 2014*a* ▸), (**II**) (WOPKAP; Nagalakshmi *et al.*, 2014*b* ▸) and (**III**) (XODQOY; Nagalakshmi *et al.*, 2014*c* ▸), showing very similar conformation of the mol­ecular core.

In the crystal of (**I**), N—H⋯O hydrogen bonds lead to the formation of chains along the *c*-axis direction. Within the chains there are further N—H⋯O and C—H⋯O hydrogen bonds enclosing 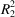 (14) ring motifs. The chains are linked *via* N—H⋯O and C—H⋯O hydrogen bonds involving the dimethyl sulfoxide solvent mol­ecule which acts as both an acceptor and a donor.

In (**II**), the asymmetric unit contains two independent mol­ecules (*A* and *B*) having similar conformations. In the crystal, mol­ecules are linked by N—H⋯O hydrogen bonds, forming chains along the *a* axis which enclose 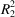 (16) ring motifs. The rings are linked by weak N—H⋯O and C—H⋯O hydrogen bonds, and C—H⋯π inter­actions, forming sheets lying parallel to the (001) plane.

In (**III**), two intra­molecular N—H⋯O hydrogen bonds are formed, each closing an *S*(6) loop. In the crystal, strong N—H⋯O hydrogen bonds lead to the formation of zigzag chains along the *c* axis. These are consolidated in the 3D crystal packing by weak N—H⋯O hydrogen bonding, as well as by C—H⋯O, C—H⋯Br and C—H⋯π inter­actions.

## 5. Synthesis and crystallization

**Figure 5:**
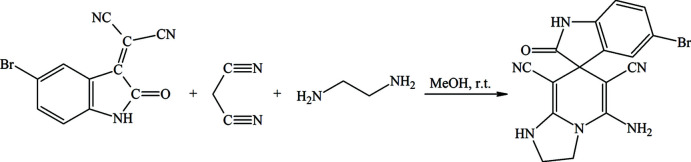
The synthesis of 5-amino-5′-bromo-2′-oxo-2,3-di­hydro-1*H*-spiro­[imidazo[1,2-*a*]pyridine-7,3′-indoline]-6,8-dicarbo­nitrile by a reported procedure (Magerramov *et al.*, 2018 ▸).

To a solution of 2-(5-bromo-2-oxoindolin-3-yl­idene)malono­nitrile (1.4 g, 5.1 mmol), which was previously prepared by a known procedure (Negar *et al.*, 2012 ▸), and malono­nitrile (0.34 g, 5.2 mmol) in methanol (25 ml), ethyl­enedi­amine (0.31 g, 5.2 mmol) was added and the mixture was stirred at room temperature for 72 h (Fig. 5 ▸). Methanol (15 ml) was removed from the reaction mixture, which was left overnight. The precipitated crystals were separated by filtration and recrystallized from an ethanol–water (1:1 *v*/*v*) solution (yield 69%; m.p. 479–480 K). Single crystals of (1) were grown from DMSO solution.

^1^H NMR (300 MHz, DMSO-*d*_6_, ppm): δ 3.50 (*t*, 4H, 2CH_2_N), 6.61 (*s*, 2H, NH_2_), 6.78 (*d*, 1H, Ar-H, ^3^ *J*_H-H_ = 7.8 Hz), 7.35 (*s*, 1H, Ar-H), 7.37 (*d*, 1H, Ar-H, ^3^ *J*_H-H_ = 7.8 Hz), 7.73 (*s*, H, NH), 10.44 (*s*, H, NH). ^13^C NMR (75 MHz, DMSO-*d*_6_, ppm): δ 42.46 (CH_2_N), 45.15 (CH_2_N), 51.24 (C_quat_), 51.71 (=C_quat_), 54.69 (=C_quat_), 112.02 (CH_arom_), 114.43 (Br—C_arom_), 119.63 (CN), 120.15 (CN), 128.02 (CH_arom_), 131.90 (CH_arom_), 137.83 (C_arom_), 140.80 (C_arom_), 152.19 (=C_quat_), 154.76 (=C_quat_), 179.67 (O=C).

## 6. Refinement

Crystal data, data collection and structure refinement details are summarized in Table 2 ▸. The H atoms were included in calculated positions and treated as riding atoms; N—H = 0.90 Å with *U*_iso_(H) = 1.2*U*_eq_(N), and C—H = 0.95–0.99 Å with *U*_iso_(H) = 1.2 or 1.5*U*_eq_(C). Both DMSO solvent mol­ecules are disordered over two positions, with final occupancies of 0.90:0.10 for the first and 0.95:0.05 for the second mol­ecule. In the first disordered DMSO molecule, the C17*B* and C18*B* atoms of the minor component were refined isotropically. The disordered atoms O2*A*/O2*B*, O3*A*/O3*B*, C19*A*/C19*B* and C20*A*/C20*B* were refined with anisotropic displacement parameters, constrained to be the same for both components. The S—C and S—O bond lengths in both disordered DMSO mol­ecules were restrained to similarity.

## Supplementary Material

Crystal structure: contains datablock(s) I, global. DOI: 10.1107/S2056989022004741/zv2013sup1.cif

Structure factors: contains datablock(s) I. DOI: 10.1107/S2056989022004741/zv2013Isup2.hkl

CCDC reference: 2170241

Additional supporting information:  crystallographic information; 3D view; checkCIF report

## Figures and Tables

**Table 1:** Hydrogen-bond geometry (Å, °)

*D*—H⋯*A*	*D*—H	H⋯*A*	*D*⋯*A*	*D*—H⋯*A*
N1—H1⋯O2*A* ^i^	0.90	1.98	2.855 (4)	165
N1—H1⋯O2*B* ^i^	0.90	2.00	2.87 (4)	160
N2—H2⋯O2*A* ^ii^	0.90	1.91	2.793 (5)	166
N2—H2⋯O2*B* ^ii^	0.90	1.94	2.82 (5)	166
N5—H5*A*⋯O3*A* ^iii^	0.90	2.20	3.034 (3)	155
N5—H5*B*⋯O3*A*	0.90	2.06	2.918 (3)	160
C2—H2*B*⋯N9^iv^	0.99	2.59	3.469 (4)	148
C19*A*—H19*A*⋯N9	0.98	2.41	3.114 (5)	128
C19*A*—H19*C*⋯O1^v^	0.98	2.52	3.392 (4)	148
C20*A*—H20*B*⋯O1^v^	0.98	2.46	3.359 (4)	152

Symmetry codes: (i) 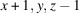 ; (ii) 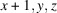 ; (iii) 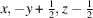 ; (iv) 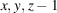 ; (v) 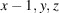 .

**Table 2:** Experimental details

Crystal data	
Chemical formula	C_16_H_11_BrN_6_O·2C_2_H_6_OS
*M*_r_	539.47
Crystal system, space group	Monoclinic, *P*2_1_/*c*
Temperature (K)	100
*a*, *b*, *c* (Å)	10.3940 (1), 26.2421 (2), 8.9860 (1)
β (°)	108.056 (1)
*V* (Å^3^)	2330.32 (4)
*Z*	4
Radiation type	Cu *K*α
μ (mm^−1^)	4.38
Crystal size (mm)	0.05 × 0.03 × 0.02

Data collection	
Diffractometer	Rigaku XtaLAB Synergy Dualflex HyPix
Absorption correction	Multi-scan (*CrysAlis PRO*; Rigaku OD, 2021 ▸)
*T*_min_, *T*_max_	0.793, 0.899
No. of measured, independent and observed [*I* > 2σ(*I*)] reflections	31508, 5062, 5047
*R*_int_	0.029
(sin θ/λ)_max_ (Å^−1^)	0.638

Refinement	
*R*[*F* ^2^ > 2σ(*F* ^2^)], *wR*(*F* ^2^), *S*	0.039, 0.094, 1.17
No. of reflections	5062
No. of parameters	331
No. of restraints	6
H-atom treatment	H-atom parameters constrained
Δρ_max_, Δρ_min_ (e Å^−3^)	0.63, −0.41

Computer programs: *CrysAlis PRO* (Rigaku OD, 2021 ▸), *SHELXT* (Sheldrick, 2015*a* ▸), *SHELXL* (Sheldrick, 2015*b* ▸), *ORTEP-3 for Windows* (Farrugia, 2012 ▸) and *PLATON* (Spek, 2020 ▸).

## supplementary crystallographic information

### Crystal data

**Table:**

C_16_H_11_BrN_6_O·2C_2_H_6_OS	*F*(000) = 1104
*M**_r_* = 539.47	*D*_x_ = 1.538 Mg m^−^^3^
Monoclinic, *P*2_1_/*c*	Cu *K*α radiation, λ = 1.54184 Å
*a* = 10.3940 (1) Å	Cell parameters from 27880 reflections
*b* = 26.2421 (2) Å	θ = 3.4–79.2°
*c* = 8.9860 (1) Å	µ = 4.38 mm^−^^1^
β = 108.056 (1)°	*T* = 100 K
*V* = 2330.32 (4) Å^3^	Prism, colourless
*Z* = 4	0.05 × 0.03 × 0.02 mm

### Data collection

**Table:**

Rigaku XtaLAB Synergy Dualflex HyPix diffractometer	5062 independent reflections
Radiation source: micro-focus sealed X-ray tube	5047 reflections with *I* > 2σ(*I*)
Detector resolution: 0 pixels mm^-1^	*R*_int_ = 0.029
φ and ω scans	θ_max_ = 79.4°, θ_min_ = 3.4°
Absorption correction: multi-scan (CrysAlis PRO; Rigaku OD, 2021)	*h* = −13→13
*T*_min_ = 0.793, *T*_max_ = 0.899	*k* = −31→33
31508 measured reflections	*l* = −11→11

### Refinement

**Table:**

Refinement on *F*^2^	Hydrogen site location: mixed
Least-squares matrix: full	H-atom parameters constrained
*R*[*F*^2^ > 2σ(*F*^2^)] = 0.039	*w* = 1/[σ^2^(*F*_o_^2^) + (0.0287*P*)^2^ + 4.5523*P*] where *P* = (*F*_o_^2^ + 2*F*_c_^2^)/3
*wR*(*F*^2^) = 0.094	(Δ/σ)_max_ = 0.003
*S* = 1.17	Δρ_max_ = 0.63 e Å^−^^3^
5062 reflections	Δρ_min_ = −0.41 e Å^−^^3^
331 parameters	Extinction correction: SHELXL (Sheldrick, 2015b), Fc^*^=kFc[1+0.001xFc^2^λ^3^/sin(2θ)]^-1/4^
6 restraints	Extinction coefficient: 0.00068 (7)

### Special details

**Table:**

Geometry. All esds (except the esd in the dihedral angle between two l.s. planes) are estimated using the full covariance matrix. The cell esds are taken into account individually in the estimation of esds in distances, angles and torsion angles; correlations between esds in cell parameters are only used when they are defined by crystal symmetry. An approximate (isotropic) treatment of cell esds is used for estimating esds involving l.s. planes.

### Fractional atomic coordinates and isotropic or equivalent isotropic displacement parameters (Å^2^)

**Table:**

	*x*	*y*	*z*	*U*_iso_*/*U*_eq_	Occ. (<1)
Br1	0.43785 (3)	0.56355 (2)	0.19141 (3)	0.02651 (10)	
O1	0.84018 (19)	0.31921 (7)	0.4894 (2)	0.0262 (4)	
N1	0.7688 (2)	0.33596 (8)	−0.0737 (2)	0.0236 (4)	
H1	0.846974	0.349380	−0.079035	0.028*	
C1	0.7948 (2)	0.36248 (9)	0.4575 (3)	0.0195 (5)	
N2	0.8234 (2)	0.40340 (8)	0.5544 (2)	0.0223 (4)	
H2	0.883850	0.403189	0.650976	0.027*	
C2	0.6818 (3)	0.30219 (12)	−0.1925 (3)	0.0311 (6)	
H2A	0.730367	0.270499	−0.201996	0.037*	
H2B	0.651308	0.319271	−0.295753	0.037*	
C3	0.5622 (3)	0.29063 (10)	−0.1340 (3)	0.0269 (6)	
H3A	0.476650	0.303812	−0.207158	0.032*	
H3B	0.553120	0.253560	−0.119462	0.032*	
N4	0.5981 (2)	0.31762 (8)	0.0156 (2)	0.0181 (4)	
C5	0.5237 (2)	0.31827 (8)	0.1184 (3)	0.0162 (4)	
N5	0.4101 (2)	0.28990 (8)	0.0779 (2)	0.0206 (4)	
H5A	0.387282	0.271975	−0.011822	0.025*	
H5B	0.357158	0.288865	0.140338	0.025*	
C6	0.5691 (2)	0.34678 (9)	0.2534 (3)	0.0175 (4)	
C7	0.6955 (2)	0.37927 (9)	0.2964 (3)	0.0164 (4)	
C8	0.7672 (2)	0.37480 (9)	0.1734 (3)	0.0170 (4)	
C8A	0.7175 (2)	0.34454 (9)	0.0441 (3)	0.0169 (4)	
C9	0.4989 (3)	0.34610 (9)	0.3650 (3)	0.0227 (5)	
N9	0.4511 (3)	0.34703 (10)	0.4650 (3)	0.0313 (5)	
C10	0.8877 (3)	0.40296 (9)	0.1953 (3)	0.0219 (5)	
N10	0.9860 (3)	0.42647 (10)	0.2186 (3)	0.0321 (5)	
C11	0.7468 (2)	0.44627 (9)	0.4847 (3)	0.0198 (5)	
C12	0.6681 (2)	0.43415 (9)	0.3327 (3)	0.0173 (4)	
C13	0.5776 (2)	0.46901 (9)	0.2424 (3)	0.0187 (5)	
H13	0.523468	0.461160	0.138649	0.022*	
C14	0.5690 (2)	0.51605 (9)	0.3097 (3)	0.0208 (5)	
C15	0.6498 (3)	0.52928 (10)	0.4587 (3)	0.0244 (5)	
H15	0.642919	0.562266	0.499107	0.029*	
C16	0.7414 (3)	0.49383 (10)	0.5490 (3)	0.0243 (5)	
H16	0.798109	0.502105	0.651194	0.029*	
S1A	0.13352 (7)	0.39460 (3)	0.95312 (8)	0.02416 (16)	0.9
O2A	−0.0128 (3)	0.38792 (11)	0.8623 (5)	0.0249 (7)	0.9
C17A	0.2226 (3)	0.38206 (13)	0.8160 (4)	0.0335 (7)	0.9
H17A	0.214127	0.345875	0.787662	0.050*	0.9
H17B	0.318415	0.390664	0.863195	0.050*	0.9
H17C	0.184208	0.402768	0.721890	0.050*	0.9
C18A	0.1618 (4)	0.46142 (14)	0.9724 (5)	0.0453 (9)	0.9
H18A	0.126943	0.477575	0.869331	0.068*	0.9
H18B	0.259115	0.468053	1.015790	0.068*	0.9
H18C	0.115059	0.475458	1.042574	0.068*	0.9
S1B	0.0823 (6)	0.4388 (2)	0.9322 (8)	0.0235 (12)	0.1
O2B	−0.024 (3)	0.3988 (13)	0.874 (6)	0.0249 (7)	0.1
C17B	0.224 (2)	0.4099 (11)	1.073 (3)	0.035 (6)*	0.1
H17D	0.252431	0.379726	1.026836	0.053*	0.1
H17E	0.198648	0.399764	1.165015	0.053*	0.1
H17F	0.299037	0.434301	1.104281	0.053*	0.1
C18B	0.150 (4)	0.4529 (15)	0.778 (3)	0.048 (8)*	0.1
H18D	0.168102	0.421089	0.730947	0.072*	0.1
H18E	0.234154	0.472179	0.819275	0.072*	0.1
H18F	0.084401	0.473284	0.697880	0.072*	0.1
S2A	0.11696 (8)	0.28805 (3)	0.26820 (8)	0.0263 (2)	0.948 (2)
O3A	0.24507 (19)	0.25919 (7)	0.2727 (2)	0.0245 (4)	0.95
C19A	0.1529 (3)	0.32357 (12)	0.4465 (4)	0.0286 (7)	0.95
H19A	0.214320	0.351728	0.444556	0.043*	0.95
H19B	0.195632	0.301184	0.535359	0.043*	0.95
H19C	0.068477	0.337256	0.457128	0.043*	0.95
C20A	0.0076 (3)	0.24245 (15)	0.3136 (6)	0.0422 (11)	0.95
H20A	−0.027573	0.219286	0.224693	0.063*	0.95
H20B	−0.067876	0.260108	0.334906	0.063*	0.95
H20C	0.058216	0.222893	0.406131	0.063*	0.95
S2B	0.0451 (16)	0.2982 (5)	0.2263 (16)	0.0263 (2)	0.052 (2)
O3B	0.165 (3)	0.2865 (13)	0.169 (4)	0.0245 (4)	0.05
C19B	0.149 (6)	0.306 (3)	0.425 (3)	0.0286 (7)	0.05
H19D	0.244796	0.302777	0.430774	0.043*	0.05
H19E	0.127063	0.279408	0.490034	0.043*	0.05
H19F	0.133444	0.339513	0.463042	0.043*	0.05
C20B	0.045 (10)	0.240 (2)	0.326 (11)	0.0422 (11)	0.05
H20D	−0.020108	0.241711	0.385397	0.063*	0.05
H20E	0.135442	0.233028	0.398604	0.063*	0.05
H20F	0.018655	0.211771	0.250113	0.063*	0.05

### Atomic displacement parameters (Å^2^)

**Table:**

	*U*^11^	*U*^22^	*U*^33^	*U*^12^	*U*^13^	*U*^23^
Br1	0.02864 (16)	0.01708 (14)	0.03326 (17)	0.00539 (10)	0.00878 (11)	0.00476 (10)
O1	0.0292 (10)	0.0233 (9)	0.0252 (9)	0.0092 (7)	0.0069 (8)	0.0058 (7)
N1	0.0255 (11)	0.0263 (11)	0.0216 (10)	−0.0070 (9)	0.0111 (9)	−0.0061 (8)
C1	0.0192 (11)	0.0212 (11)	0.0186 (11)	0.0012 (9)	0.0067 (9)	0.0025 (9)
N2	0.0216 (10)	0.0247 (11)	0.0165 (9)	0.0033 (8)	−0.0002 (8)	0.0011 (8)
C2	0.0280 (14)	0.0391 (16)	0.0275 (13)	−0.0081 (12)	0.0106 (11)	−0.0137 (12)
C3	0.0325 (14)	0.0252 (13)	0.0228 (12)	−0.0088 (11)	0.0085 (11)	−0.0084 (10)
N4	0.0179 (9)	0.0202 (10)	0.0161 (9)	−0.0034 (8)	0.0054 (8)	−0.0035 (8)
C5	0.0178 (10)	0.0130 (10)	0.0179 (10)	0.0018 (8)	0.0056 (9)	0.0032 (8)
N5	0.0214 (10)	0.0218 (10)	0.0192 (10)	−0.0040 (8)	0.0074 (8)	−0.0034 (8)
C6	0.0196 (11)	0.0148 (10)	0.0188 (11)	−0.0009 (9)	0.0068 (9)	−0.0007 (8)
C7	0.0185 (11)	0.0136 (10)	0.0164 (10)	0.0000 (8)	0.0044 (9)	−0.0004 (8)
C8	0.0196 (11)	0.0144 (10)	0.0169 (11)	−0.0012 (8)	0.0054 (9)	0.0008 (8)
C8A	0.0176 (11)	0.0136 (10)	0.0192 (11)	0.0012 (8)	0.0052 (9)	0.0021 (8)
C9	0.0247 (12)	0.0192 (11)	0.0240 (12)	−0.0048 (9)	0.0073 (10)	−0.0059 (9)
N9	0.0330 (12)	0.0352 (13)	0.0300 (12)	−0.0105 (10)	0.0162 (10)	−0.0130 (10)
C10	0.0263 (13)	0.0197 (11)	0.0204 (11)	−0.0026 (10)	0.0084 (10)	−0.0031 (9)
N10	0.0312 (12)	0.0331 (13)	0.0339 (13)	−0.0126 (10)	0.0130 (10)	−0.0100 (10)
C11	0.0201 (11)	0.0202 (11)	0.0168 (11)	−0.0012 (9)	0.0025 (9)	0.0007 (9)
C12	0.0187 (11)	0.0159 (11)	0.0171 (11)	−0.0023 (8)	0.0052 (9)	−0.0025 (8)
C13	0.0205 (11)	0.0169 (11)	0.0170 (11)	−0.0019 (9)	0.0037 (9)	−0.0004 (9)
C14	0.0204 (11)	0.0170 (11)	0.0251 (12)	0.0004 (9)	0.0069 (10)	0.0029 (9)
C15	0.0307 (13)	0.0180 (11)	0.0258 (13)	−0.0031 (10)	0.0106 (11)	−0.0058 (10)
C16	0.0273 (13)	0.0243 (12)	0.0195 (11)	−0.0049 (10)	0.0046 (10)	−0.0065 (10)
S1A	0.0207 (3)	0.0278 (4)	0.0216 (3)	−0.0033 (3)	0.0030 (3)	0.0009 (3)
O2A	0.0197 (10)	0.0340 (18)	0.0191 (12)	−0.0052 (12)	0.0032 (8)	0.0003 (13)
C17A	0.0303 (16)	0.0348 (17)	0.0396 (18)	−0.0050 (13)	0.0172 (14)	0.0012 (14)
C18A	0.046 (2)	0.0296 (18)	0.064 (3)	−0.0077 (17)	0.0228 (19)	−0.0129 (17)
S1B	0.023 (3)	0.011 (3)	0.041 (3)	0.004 (2)	0.017 (3)	0.005 (2)
O2B	0.0197 (10)	0.0340 (18)	0.0191 (12)	−0.0052 (12)	0.0032 (8)	0.0003 (13)
S2A	0.0283 (4)	0.0275 (3)	0.0226 (3)	0.0065 (3)	0.0074 (3)	0.0015 (3)
O3A	0.0256 (10)	0.0235 (9)	0.0263 (10)	0.0008 (8)	0.0108 (8)	−0.0013 (7)
C19A	0.0278 (14)	0.0235 (15)	0.0378 (16)	−0.0057 (13)	0.0148 (12)	−0.0156 (12)
C20A	0.028 (2)	0.0434 (18)	0.062 (2)	−0.0157 (15)	0.023 (2)	−0.0283 (17)
S2B	0.0283 (4)	0.0275 (3)	0.0226 (3)	0.0065 (3)	0.0074 (3)	0.0015 (3)
O3B	0.0256 (10)	0.0235 (9)	0.0263 (10)	0.0008 (8)	0.0108 (8)	−0.0013 (7)
C19B	0.0278 (14)	0.0235 (15)	0.0378 (16)	−0.0057 (13)	0.0148 (12)	−0.0156 (12)
C20B	0.028 (2)	0.0434 (18)	0.062 (2)	−0.0157 (15)	0.023 (2)	−0.0283 (17)

### Geometric parameters (Å, º)

**Table:**

Br1—C14	1.909 (2)	C16—H16	0.9500
O1—C1	1.229 (3)	S1A—O2A	1.497 (3)
N1—C8A	1.344 (3)	S1A—C18A	1.778 (4)
N1—C2	1.464 (3)	S1A—C17A	1.787 (3)
N1—H1	0.9000	C17A—H17A	0.9800
C1—N2	1.356 (3)	C17A—H17B	0.9800
C1—C7	1.559 (3)	C17A—H17C	0.9800
N2—C11	1.408 (3)	C18A—H18A	0.9800
N2—H2	0.8999	C18A—H18B	0.9800
C2—C3	1.523 (4)	C18A—H18C	0.9800
C2—H2A	0.9900	S1B—O2B	1.497 (4)
C2—H2B	0.9900	S1B—C18B	1.777 (5)
C3—N4	1.462 (3)	S1B—C17B	1.787 (5)
C3—H3A	0.9900	C17B—H17D	0.9800
C3—H3B	0.9900	C17B—H17E	0.9800
N4—C5	1.377 (3)	C17B—H17F	0.9800
N4—C8A	1.381 (3)	C18B—H18D	0.9800
C5—N5	1.347 (3)	C18B—H18E	0.9800
C5—C6	1.378 (3)	C18B—H18F	0.9800
N5—H5A	0.8996	S2A—O3A	1.521 (2)
N5—H5B	0.8999	S2A—C20A	1.782 (3)
C6—C9	1.412 (3)	S2A—C19A	1.790 (3)
C6—C7	1.513 (3)	C19A—H19A	0.9800
C7—C8	1.517 (3)	C19A—H19B	0.9800
C7—C12	1.523 (3)	C19A—H19C	0.9800
C8—C8A	1.369 (3)	C20A—H20A	0.9800
C8—C10	1.414 (3)	C20A—H20B	0.9800
C9—N9	1.154 (4)	C20A—H20C	0.9800
C10—N10	1.156 (4)	S2B—O3B	1.522 (4)
C11—C16	1.384 (4)	S2B—C20B	1.782 (5)
C11—C12	1.394 (3)	S2B—C19B	1.790 (4)
C12—C13	1.381 (3)	C19B—H19D	0.9800
C13—C14	1.390 (3)	C19B—H19E	0.9800
C13—H13	0.9500	C19B—H19F	0.9800
C14—C15	1.386 (4)	C20B—H20D	0.9800
C15—C16	1.397 (4)	C20B—H20E	0.9800
C15—H15	0.9500	C20B—H20F	0.9800
			
C8A—N1—C2	111.7 (2)	C11—C16—H16	121.1
C8A—N1—H1	124.1	C15—C16—H16	121.1
C2—N1—H1	124.2	O2A—S1A—C18A	106.15 (19)
O1—C1—N2	126.2 (2)	O2A—S1A—C17A	105.0 (2)
O1—C1—C7	125.0 (2)	C18A—S1A—C17A	98.23 (18)
N2—C1—C7	108.8 (2)	S1A—C17A—H17A	109.5
C1—N2—C11	111.5 (2)	S1A—C17A—H17B	109.5
C1—N2—H2	124.2	H17A—C17A—H17B	109.5
C11—N2—H2	124.3	S1A—C17A—H17C	109.5
N1—C2—C3	104.8 (2)	H17A—C17A—H17C	109.5
N1—C2—H2A	110.8	H17B—C17A—H17C	109.5
C3—C2—H2A	110.8	S1A—C18A—H18A	109.5
N1—C2—H2B	110.8	S1A—C18A—H18B	109.5
C3—C2—H2B	110.8	H18A—C18A—H18B	109.5
H2A—C2—H2B	108.9	S1A—C18A—H18C	109.5
N4—C3—C2	102.6 (2)	H18A—C18A—H18C	109.5
N4—C3—H3A	111.3	H18B—C18A—H18C	109.5
C2—C3—H3A	111.3	O2B—S1B—C18B	107 (2)
N4—C3—H3B	111.3	O2B—S1B—C17B	108 (2)
C2—C3—H3B	111.3	C18B—S1B—C17B	101.7 (16)
H3A—C3—H3B	109.2	S1B—C17B—H17D	109.5
C5—N4—C8A	121.9 (2)	S1B—C17B—H17E	109.5
C5—N4—C3	125.9 (2)	H17D—C17B—H17E	109.5
C8A—N4—C3	112.2 (2)	S1B—C17B—H17F	109.5
N5—C5—N4	116.1 (2)	H17D—C17B—H17F	109.5
N5—C5—C6	124.8 (2)	H17E—C17B—H17F	109.5
N4—C5—C6	119.1 (2)	S1B—C18B—H18D	109.5
C5—N5—H5A	119.8	S1B—C18B—H18E	109.5
C5—N5—H5B	120.2	H18D—C18B—H18E	109.5
H5A—N5—H5B	120.0	S1B—C18B—H18F	109.5
C5—C6—C9	120.5 (2)	H18D—C18B—H18F	109.5
C5—C6—C7	124.4 (2)	H18E—C18B—H18F	109.5
C9—C6—C7	115.1 (2)	O3A—S2A—C20A	105.94 (15)
C6—C7—C8	110.72 (19)	O3A—S2A—C19A	107.28 (13)
C6—C7—C12	112.51 (19)	C20A—S2A—C19A	96.65 (19)
C8—C7—C12	113.31 (19)	S2A—C19A—H19A	109.5
C6—C7—C1	110.56 (19)	S2A—C19A—H19B	109.5
C8—C7—C1	108.74 (19)	H19A—C19A—H19B	109.5
C12—C7—C1	100.51 (18)	S2A—C19A—H19C	109.5
C8A—C8—C10	120.4 (2)	H19A—C19A—H19C	109.5
C8A—C8—C7	121.5 (2)	H19B—C19A—H19C	109.5
C10—C8—C7	118.2 (2)	S2A—C20A—H20A	109.5
N1—C8A—C8	128.9 (2)	S2A—C20A—H20B	109.5
N1—C8A—N4	108.7 (2)	H20A—C20A—H20B	109.5
C8—C8A—N4	122.3 (2)	S2A—C20A—H20C	109.5
N9—C9—C6	174.4 (3)	H20A—C20A—H20C	109.5
N10—C10—C8	177.5 (3)	H20B—C20A—H20C	109.5
C16—C11—C12	121.8 (2)	O3B—S2B—C20B	97 (4)
C16—C11—N2	128.7 (2)	O3B—S2B—C19B	93 (3)
C12—C11—N2	109.4 (2)	C20B—S2B—C19B	72 (4)
C13—C12—C11	120.7 (2)	S2B—C19B—H19D	109.5
C13—C12—C7	129.7 (2)	S2B—C19B—H19E	109.5
C11—C12—C7	109.5 (2)	H19D—C19B—H19E	109.5
C12—C13—C14	117.1 (2)	S2B—C19B—H19F	109.5
C12—C13—H13	121.4	H19D—C19B—H19F	109.5
C14—C13—H13	121.4	H19E—C19B—H19F	109.5
C15—C14—C13	122.8 (2)	S2B—C20B—H20D	109.5
C15—C14—Br1	119.25 (19)	S2B—C20B—H20E	109.5
C13—C14—Br1	117.94 (18)	H20D—C20B—H20E	109.5
C14—C15—C16	119.6 (2)	S2B—C20B—H20F	109.5
C14—C15—H15	120.2	H20D—C20B—H20F	109.5
C16—C15—H15	120.2	H20E—C20B—H20F	109.5
C11—C16—C15	117.8 (2)		
			
O1—C1—N2—C11	−175.7 (2)	C2—N1—C8A—C8	−178.1 (3)
C7—C1—N2—C11	5.0 (3)	C2—N1—C8A—N4	0.9 (3)
C8A—N1—C2—C3	1.0 (3)	C10—C8—C8A—N1	0.9 (4)
N1—C2—C3—N4	−2.3 (3)	C7—C8—C8A—N1	−179.2 (2)
C2—C3—N4—C5	−178.4 (2)	C10—C8—C8A—N4	−178.0 (2)
C2—C3—N4—C8A	3.0 (3)	C7—C8—C8A—N4	1.9 (3)
C8A—N4—C5—N5	−179.2 (2)	C5—N4—C8A—N1	178.8 (2)
C3—N4—C5—N5	2.4 (3)	C3—N4—C8A—N1	−2.6 (3)
C8A—N4—C5—C6	0.3 (3)	C5—N4—C8A—C8	−2.1 (3)
C3—N4—C5—C6	−178.2 (2)	C3—N4—C8A—C8	176.5 (2)
N5—C5—C6—C9	2.8 (4)	C1—N2—C11—C16	175.8 (3)
N4—C5—C6—C9	−176.6 (2)	C1—N2—C11—C12	−2.6 (3)
N5—C5—C6—C7	−178.9 (2)	C16—C11—C12—C13	−2.4 (4)
N4—C5—C6—C7	1.7 (3)	N2—C11—C12—C13	176.2 (2)
C5—C6—C7—C8	−1.8 (3)	C16—C11—C12—C7	−179.5 (2)
C9—C6—C7—C8	176.6 (2)	N2—C11—C12—C7	−1.0 (3)
C5—C6—C7—C12	126.2 (2)	C6—C7—C12—C13	−55.6 (3)
C9—C6—C7—C12	−55.4 (3)	C8—C7—C12—C13	70.9 (3)
C5—C6—C7—C1	−122.3 (2)	C1—C7—C12—C13	−173.2 (2)
C9—C6—C7—C1	56.1 (3)	C6—C7—C12—C11	121.2 (2)
O1—C1—C7—C6	56.6 (3)	C8—C7—C12—C11	−112.3 (2)
N2—C1—C7—C6	−124.1 (2)	C1—C7—C12—C11	3.6 (2)
O1—C1—C7—C8	−65.2 (3)	C11—C12—C13—C14	0.0 (4)
N2—C1—C7—C8	114.1 (2)	C7—C12—C13—C14	176.5 (2)
O1—C1—C7—C12	175.6 (2)	C12—C13—C14—C15	2.3 (4)
N2—C1—C7—C12	−5.1 (2)	C12—C13—C14—Br1	−176.74 (18)
C6—C7—C8—C8A	0.0 (3)	C13—C14—C15—C16	−2.1 (4)
C12—C7—C8—C8A	−127.5 (2)	Br1—C14—C15—C16	176.9 (2)
C1—C7—C8—C8A	121.6 (2)	C12—C11—C16—C15	2.5 (4)
C6—C7—C8—C10	179.8 (2)	N2—C11—C16—C15	−175.7 (2)
C12—C7—C8—C10	52.4 (3)	C14—C15—C16—C11	−0.3 (4)
C1—C7—C8—C10	−58.5 (3)		

### Hydrogen-bond geometry (Å, º)

**Table:**

*D*—H···*A*	*D*—H	H···*A*	*D*···*A*	*D*—H···*A*
N1—H1···O2*A*^i^	0.90	1.98	2.855 (4)	165
N1—H1···O2*B*^i^	0.90	2.00	2.87 (4)	160
N2—H2···O2*A*^ii^	0.90	1.91	2.793 (5)	166
N2—H2···O2*B*^ii^	0.90	1.94	2.82 (5)	166
N5—H5*A*···O3*A*^iii^	0.90	2.20	3.034 (3)	155
N5—H5*B*···O3*A*	0.90	2.06	2.918 (3)	160
C2—H2*B*···N9^iv^	0.99	2.59	3.469 (4)	148
C19*A*—H19*A*···N9	0.98	2.41	3.114 (5)	128
C19*A*—H19*C*···O1^v^	0.98	2.52	3.392 (4)	148
C20*A*—H20*B*···O1^v^	0.98	2.46	3.359 (4)	152

Symmetry codes: (i) *x*+1, *y*, *z*−1; (ii) *x*+1, *y*, *z*; (iii) *x*, −*y*+1/2, *z*−1/2; (iv) *x*, *y*, *z*−1; (v) *x*−1, *y*, *z*.

## References

[bb1] Abdelhamid, A. A., Mohamed, S. K., Khalilov, A. N., Gurbanov, A. V. & Ng, S. W. (2011). *Acta Cryst.* E**67**, o744. 10.1107/S1600536811006969 PMC3051986 21522483

[bb2] Bernstein, J., Davis, R. E., Shimoni, L. & Chang, N.-L. (1995). *Angew. Chem. Int. Ed. Engl.* **34**, 1555–1573.

[bb3] Farrugia, L. J. (2012). *J. Appl. Cryst.* **45**, 849–854.

[bb4] Groom, C. R., Bruno, I. J., Lightfoot, M. P. & Ward, S. C. (2016). *Acta Cryst.* B**72**, 171–179. 10.1107/S2052520616003954 PMC4822653 27048719

[bb5] Han, F., Shioda, N., Moriguchi, S., Yamamoto, Y., Raie, A. Y. A., Yamaguchi, Y., Hino, M. & Fukunaga, K. (2008). *J. Pharmacol. Exp. Ther.* **326**, 127–134. 10.1124/jpet.108.137471 18388258

[bb6] Jun, J., Juan, L., Xinhua, L., Hongxin, L., Haiyan, W. & Hong-Ping, X. (2019). *Synlett*, **30**, 1241–1245.

[bb7] Khalilov, A. N., Tüzün, B., Taslimi, P., Tas, A., Tuncbilek, Z. & Cakmak, N. K. (2021). *J. Mol. Liq.* **344**, 117761.

[bb8] Lu, L., Deyan, W., Xiangmin, L., Sinan, W., Hao, L., Jian, L. & Wei, W. (2012). *Chem. Commun.* **48**, 1692–1694.

[bb9] Magerramov, A. M., Nagiev, F. N., Mamedova, G. Z. Kh. A., Asadov, Kh. A. & Mamedov, I. G. (2018). *Russ. J. Org. Chem.* **54**, 1713–1715.

[bb10] Mamedov, I., Naghiyev, F., Maharramov, A., Uwangue, O., Farewell, A., Sunnerhagen, P. & Erdelyi, M. (2020). *Mendeleev Commun.* **30**, 498–499.

[bb11] Nagalakshmi, R. A., Suresh, J., Sivakumar, S., Kumar, R. R. & Lakshman, P. L. N. (2014*a*). *Acta Cryst.* E**70**, o604–o605. 10.1107/S1600536814008800 PMC4011289 24860398

[bb12] Nagalakshmi, R. A., Suresh, J., Sivakumar, S., Kumar, R. R. & Lakshman, P. L. N. (2014*b*). *Acta Cryst.* E**70**, o971–o972. 10.1107/S1600536814017486 PMC4186207 25309286

[bb13] Nagalakshmi, R. A., Suresh, J., Sivakumar, S., Kumar, R. R. & Lakshman, P. L. N. (2014*c*). *Acta Cryst.* E**70**, o816–o817. 10.1107/S1600536814014391 PMC4120621 25161594

[bb14] Naghiyev, F. N., Cisterna, J., Khalilov, A. N., Maharramov, A. M., Askerov, R. K., Asadov, K. A., Mamedov, I. G., Salmanli, K. S., Cárdenas, A. & Brito, I. (2020). *Molecules*, **25**, 2235–2248. 10.3390/molecules25092235 PMC7248728 32397450

[bb15] Naghiyev, F. N., Grishina, M. M., Khrustalev, V. N., Khalilov, A. N., Akkurt, M., Akobirshoeva, A. A. & Mamedov, İ. G. (2021*a*). *Acta Cryst.* E**77**, 195–199. 10.1107/S2056989021000785 PMC7869549 33614153

[bb16] Naghiyev, F. N., Tereshina, T. A., Khrustalev, V. N., Akkurt, M., Khalilov, A. N., Akobirshoeva, A. A. & Mamedov, İ. G. (2021*b*). *Acta Cryst.* E**77**, 512–515. 10.1107/S2056989021003625 PMC8100277 34026255

[bb17] Negar, L., Ghodsi Mohammadi, Z., Alireza, B. & Parisa, G. (2012). *Eur. J. Chem.* **3**, 310–313.

[bb18] Rigaku OD (2021). *CrysAlis PRO*. Rigaku Oxford Diffraction Ltd, Yarnton, England.

[bb19] Samaneh, A., Homa, A. & Javad, A. (2021). *J. Chin. Chem. Soc.* **68**, 1090–1103.

[bb20] Sheldrick, G. M. (2015*a*). *Acta Cryst.* A**71**, 3–8.

[bb21] Sheldrick, G. M. (2015*b*). *Acta Cryst.* C**71**, 3–8.

[bb22] Spek, A. L. (2020). *Acta Cryst.* E**76**, 1–11. 10.1107/S2056989019016244 PMC6944088 31921444

[bb23] Turner, M. J., McKinnon, J. J., Wolff, S. K., Grimwood, D. J., Spackman, P. R., Jayatilaka, D. & Spackman, M. A. (2017). *CrystalExplorer17*. The University of Western Australia.

[bb24] Yadigarov, R. R., Khalilov, A. N., Mamedov, I. G., Nagiev, F. N., Magerramov, A. M. & Allakhverdiev, M. A. (2009). *Russ. J. Org. Chem.* **45**, 1856–1858.

[bb25] Yin, J., Khalilov, A. N., Muthupandi, P., Ladd, R. & Birman, V. B. (2020). *J. Am. Chem. Soc.* **142**, 60–63. 10.1021/jacs.9b11160 31873004

